# Factors influencing older adults’ satisfaction with caregivers’ communication

**DOI:** 10.1590/1980-5764-DN-2023-0069

**Published:** 2023-12-04

**Authors:** EL Mahjoub EL Harsi, Ouafa Izel, Abdelhafid Benksim, Mohamed Cherkaoui

**Affiliations:** 1 Cadi Ayyad University, Faculty of Sciences Semlalia, Laboratory of Pharmacology, Neurobiology, Anthropobiology and Environment, Marrakech 40000, Morocco.; 2 Regional Health Directorate, Higher Institute of Nursing Professions and Health Techniques (ISPITS-M), Nursing Care Department, Marrakech 40000, Morocco.

**Keywords:** Communication, Aged, Caregivers, Patients, Aging, Comunicação, Idoso, Cuidadores, Pacientes, Envelhecimento

## Abstract

Effective communication skills are crucial for caregivers to provide quality care and meet the unique needs of patients of all ages. However, older patients have specific communication requirements, and their satisfaction depends on several factors.

**Objective:**

This study aimed to evaluate the level of satisfaction among older adults in Marrakech, Morocco, regarding the communication skills of their caregivers, and to identify the factors influencing this satisfaction.

**Methods:**

This is a cross-sectional study conducted between March and July 2022 among 204 people aged 60 years and older who presented to the Mouhamed VI University Hospital of Marrakech, Morocco, for various care services. The older adults’ satisfaction with caregivers’ communication was assessed by the American Board of Internal Medicine (ABIM) patient satisfaction questionnaire. Sociodemographic and clinical characteristics of the participants were collected through interview and consultation of medical records. Multiple linear regression was used to determine potential factors influencing the total satisfaction score.

**Results:**

The total satisfaction score of older adults with caregiver’ communication was 2.55±0.95 and the mean scores of the lowest subscales were answering questions, greeting and listening. Analysis revealed that having visual disorders (B=-0.276±0.12; p=0.029) and receiving affective touch from caregivers (B=0.745±0.12; p=0.001) were the main factors associated with older adults’ satisfaction with caregiver’ communication.

**Conclusion:**

Older people are not sufficiently satisfied with caregivers’ communication skills, especially those with vision problems and those who have not received affective touch from caregivers. Caregivers need to be aware of the specific needs of older patients and use appropriate communication techniques.

## RESUMO.

Habilidades de comunicação eficazes são fundamentais para que os profissionais de saúde ofereçam atendimento de qualidade e atendam às necessidades específicas de pacientes de todas as idades. No entanto, os pacientes mais velhos têm necessidades específicas de comunicação, e sua satisfação depende de vários fatores.

**Objetivo:**

Este estudo teve como objetivo avaliar a satisfação da pessoa idosa com a comunicação dos cuidadores e identificar os fatores de influência.

**Métodos:**

Este é um estudo transversal realizado entre março e julho de 2022 com 204 pessoas com 60 anos ou mais que se apresentaram ao Hospital Universitário Mouhamed VI de Marrakech para receber diversos cuidados. A satisfação da pessoa idosa com a comunicação dos cu idadores foi avaliada pelo questionário de satisfação do paciente do American Board of Internal Medicine (ABIM). As características sociodemográficas e clínicas dos participantes foram coletadas por meio de entrevista e consulta aos registros médicos. A análise dos dados foi realizada com o *software Statistical Package for the Social Sciences* (SPSS), versão 25.

**Resultados:**

A pontuação total de satisfação da pessoa idosa com a comunicação do cuidador foi de 2,55±0,95, e as pontuações médias das subescalas mais baixas foram responder a perguntas, cumprimentar e ouvir. A análise revelou que ter distúrbios visuais (B=-0,276±0,12; p=0,029) e receber toque afetivo dos cuidadores (B=0,745±0,12; p=0,001) foram os principais fatores associados à satisfação dos pessoa idosa com a comunicação do cuidador.

**Conclusão:**

As pessoas idosas não estão suficientemente satisfeitas com as habilidades de comunicação dos cuidadores, especialmente aquelas com problemas de visão e as que não receberam o toque afetivo dos cuidadores. Os cuidadores precisam estar cientes das necessidades específicas dos pacientes mais velhos e usar técnicas de comunicação adequadas.

## INTRODUCTION

Aging is a natural, irreversible and inevitable physiological process that causes changes in organism structure and functions^1^ These changes can be accompanied by several health problems such as chronic diseases, reduced physical and cognitive abilities, sensory disorders, osteoarticular affections, depression, etc^2–4^.

With advancing age, the number of morbidities increases and makes older adults more fragile, hence the need for better health care to mitigate the impact of these disorders on quality of life.

Health care encompasses physical, mental and social well-being, not just the treatment of a specific disease, with the goal of providing people with global, high-quality care according to their health needs throughout their lives^5^.

Several studies have shown that the effectiveness and quality of care depends on the quality of caregiver-care recipient communication, whether nurse or physician^6–9^. Communication is the basis for establishing a good caregiver-care recipient relationship; patients expect to be greeted, listened to, and understood by their caregivers^10^, while caregivers, through successful communication with the patient, can identify symptoms, establish a diagnosis, prescribe treatment, and subsequently achieve compliance, adherence, and satisfaction with care^11–13^.

Older adults have different communication needs and desires than other age groups because they have specific linguistic, cognitive, physiological, and social problems^14,15^. This presents a challenge for healthcare providers, who need to be aware of these specificities and mobilize appropriate communication techniques in order to establish a trusting relationship with the older patient, gain their satisfaction, and then provide quality care.

Indeed, in the face of the aging population that the world is currently experiencing, the management of morbidities associated with aging and the provision of quality care constitute a major public health issue in order to preserve the quality of life of healthy older people and ensure their successful aging.

Several authors announced that older adults’ satisfaction is an important element in providing them with quality care^16–18^. Therefore, it is of interest to assess older adults’ satisfaction with caregivers’ communication and to determine the factors that need to be addressed to satisfy older adults and ensure better health care for them.

Several studies with patients of different age groups showed that caregiver-care recipient communication could be influenced by several patient-related factors, including age^19^, gender^20^, education level^19,20^, visual impairment^21^, hearing deficits^22^, speech difficulties^23^, depression^24^, dependency^25^, and cognitive disorders^26^. However, few studies have been conducted with older patients.

The objective of this study is to assess the satisfaction of older adults in the Marrakech-Safi region with caregivers’ communication and to identify influencing factors.

## METHODS

### Ethics approval and consent to participate

The study was conducted according to the ethical standards of the Declaration of Helsinki. It was approved by the University Hospital Ethics Committee of Marrakech (reference n° 26/2021). Written informed consent was obtained for all participants before the start of the study via an information sheet on the conduct of the survey. Confidentiality of information was maintained throughout the study.

### Design of the study

This is a cross-sectional study, with a quantitative mode of investigation, that aims to assess older adults’ satisfaction with caregivers’ communication and to examine influencing factors.

The target population was patients aged 60 years and older who presented to the Mohammed VI University Hospital of Marrakech (Med VI UH). This is a referral institution that includes all medical specialties and receives older persons from all the provinces of the region who come for various care services, especially since the region does not have specific geriatric services. The inclusion criteria were people aged 60 years and older who received care from caregivers in Med VI UH. The exclusion criteria were people with severe psychiatric and neurological disorders. A total of 220 individuals aged 60 and above were included in this study through non-probabilistic accidental sampling. These individuals were selected from those present during the survey period between March and July 2022 in various departments of Med VI UH. Sixteen individuals refused to complete the questionnaire. Thus, a final sample of 204 patients aged 60 and above was identified as eligible to be included in this study.

### Data collection

Data were collected through a face-to-face interview with older adults using a structured questionnaire that contained both closed-ended and multiple-choice questions, and also through consultation of the participants’ medical records. The interview was conducted by the investigator at the time of the participants’ discharge from the hospital after receiving health care from nurses or doctors. In order not to influence participants’ responses, the investigator was not a member of the Med VI UH care team and did not wear a gown during the survey.

### Variables and modalities

Older adults’ satisfaction with caregivers’ communication was assessed by the American Board of Internal Medicine (ABIM) patient satisfaction questionnaire. This is a tool that assesses six communication items, including greeting, listening, showing interest, respect, answering questions, and use of simple language by caregivers. Patients were asked to rate each of these items on a five-point Likert scale: 1=poor, 2=fair, 3=good, 4=very good and 5=excellent^27^. The total satisfaction score is the average of the scores for these six items, ranging from one for the worst score to five for the best score. The ABIM is a reliable and valid tool used in several studies and in different contexts to assess patients’ satisfaction with caregivers’ professionalism and communication skills^28,29^.

Sociodemographic data of older adults were collected through an interview that included several variables such as age, sex, origin (urban or rural), marital status (with or without spouse) and educational status (illiterate, primary, secondary or higher level). Economic level is estimated on the basis of monthly income (low: less than 3,000 Moroccan Dirham (MD), medium: between 3,000 and 6,000 MD, high: above 6,000 MD).

Data on participants’ health status were collected by consultation of medical records and self-reporting of the respondents, such as the presence of chronic disease (diabetes, hypertension, cardiovascular disease, etc.).

For the sensory variables, hearing status was assessed using the Hearing Handicap Inventory for the Elderly (HHIE-S) with ten items scored from 0 to 40. Thus, every person who has a score³10 is considered to have a hearing loss^30^. Participants were classified into two categories (with or without hearing loss). Vision status was determined by an interview and review of participants’ medical records (a person is identified as having vision impairment if he/she reports having difficulty seeing or has ocular pathologies such as diabetic retinopathy, cataract, age-related macular degeneration, or any other eye disease that affects vision).

Speech abilities were assessed by the question “Do you ever lose your thoughts and words when communicating with caregivers?”, with three response modalities: “never”, “sometimes” and “always”.

In addition, data on older adults’ perceptions of caregiver affective touch were collected through the question: “Do you benefit from caregivers’ affective touch during caregiving activities?”, with two response modalities: “yes” or “no”.

Functional abilities were measured through the Lawton scale, which assesses instrumental activities of daily living (IADL) and includes eight items: using the telephone, shopping, preparing meals, housework, laundry, taking medication, managing money, taking short trips^31^. IADL was coded into two categories:

- No dependencies related to instrumental activities of daily living; and
- Dependencies related to instrumental activities of daily living, if the participant answered *“I can’t do it at all*” or *“I have a lot of difficulty”* for one or more items.

Depressive symptoms were assessed using a 15-item version of the Geriatric Depression Scale (GDS), ranging from 0 to 15 points:

- normal: 0–5;
- moderate depression: 6–10; and
- severe depression: 11–15^32^.

### Statistical analysis

Data were examined using IBM’s Statistical Package for the Social Sciences (SPSS) version 25.Ink software. Cronbach’s alpha coefficient was utilized to assess the internal consistency of the items on the ABIM scale. A Cronbach’s alpha coefficient greater than 0.7 was considered indicative of a reliable scale. A one-sample Kolmogorov-Smirnov test was used to analyze the normality of continuous variables. Descriptive analyses were used such as frequency, mean, standard deviation, and median to describe the subscale scores and the total ABIM questionnaire score. Given that the normality hypothesis is not acceptable, the nonparametric Kruskal-Wallis test was used to examine the association between the total score on the ABIM satisfaction scale and sociodemographic, economic, and health characteristics of older adults. If the results of the Kruskal-Wallis test showed significant associations, the Mann-Whitney U test was used to make a comparison between pairs. Multiple linear regression was used to determine potential factors influencing the total satisfaction score, using the factor(s) identified as significant in the univariate analysis. Statistical significance was defined as p<0.05.

## RESULTS

A total of 204 individuals aged 60 years and older were included in this study, with a mean age of 67.75±6.55 years. Most participants were female (54.9%), urban (59.3%), and married (70.1%). Regarding educational status, 49.5% were illiterate, 19.1% had primary education, 23.5% had secondary education and 7.8% had higher education. In terms of economic status, the majority of participants had low income (75.5%).

The test of the reliability of the ABIM questionnaire showed that the internal consistency of the overall scale was very satisfactory (coefficient alpha=0.94). Cro bach’s coefficients were greater than 0.7 for all subscales. They were for greeting (0.79), listening (0.81), showing interest (0.87), respect (0.77), answering questions (0.82), and using simple language (0.81).

Descriptive analysis showed that the mean total satisfaction score of older adults with caregivers’ communication was 2.55±0.95. The mean scores of the lowest subscales were for answering questions (2.44±1.10), greeting (2.51±1.03), listening (2.54±1.13), and interest (2.55±1.06) (Table 1).

**Table 1:** Composite and subscale scores of older adults’ satisfaction with caregivers’ communication.

Communication skills	Mean±SD	Median
Greeting	2.51±1.03	2.00
Respect	2.63±1.07	3.00
Listening	2.54±1.13	2.00
Interest	2.55±1.06	2.00
Answering and encouraging questions	2.44±1.10	2.00
Simple language	2.65±1.18	2.00
Total score	2.55±0.95	2.33

Abbreviation: SD, standard deviation.

Table 2 presents the total satisfaction score of older adults with caregivers’ communication according to sociodemographic and economic characteristics. According to this table, there is no significant relationship between satisfaction and age, sex, origin, marital status, education level and income (p³0.05).

**Table 2:** Total satisfaction score of older adults with caregivers’ communication by sociodemographic and economic characteristics.

Variables		n (%)	Mean±SD	p-value
Age (years)				
	60 to 69	137 (67.2)	2.58±1.03	0.549
	³70	67 (32.8)	2.50±0.96	
Sex				
	Male	92 (45.1)	2.59±1.05	0.917
	Female	112 (54.9)	2.52±0.86	
Origin				
	Urban	121 (59.3)	2.49±0.87	0.472
	Rural	83 (40.7)	2.63±1.05	
Marital status				
	With spouse	143 (70.1)	2.58±1.03	0.929
	Without spouse	61 (29.9)	2.48±0.73	
Education				
	Illiterate	101 (46.5)	2.61±0.96	0.077
	Primary	39 (19.1)	2.38±0.96	
	Secondary	48 (23.5)	2.430.93	
	Higher	16 (7.5)	3.00±1.00	
Income				
	Low	154 (75.5)	2.540.96	0.809
	Medium	46 (22.5)	2.60±0.95	
	High	4 (2)	2.50±0.75	

Abbreviation: SD, standard deviation.

For health characteristics, the bivariate analysis showed that the mean satisfaction score was lower in people with hearing impairment and in those with vision disorders compared to people without these impairments, with (2.29 *vs.* 2.71) and (2.39 *vs.* 2.85), respectively. However, the analysis did not find an association between the mean satisfaction score and chronic diseases and dependence on instrumental activities of daily living (p³0.05) (Table 3).

**Table 3:** Total satisfaction score of older adults with caregivers’ communication by health characteristics.

Variables			Mean±SD	p-value
Chronic diseases				
	Yes	117 (57.4)	2.57±0.95	0.835
	None	87 (42.6)	2.53±0.96	
Hearing impairment				
	Yes	77 (37.7)	2.29±0.99	0.001
	None	127 (62.3)	2.71±0.90	
Visuel impairment				
	Yes	133 (65.2)	2.39±0.88	0.001
	None	71 (34.8)	2.85±1.01	
Lost words and thoughts				
	Never	100 (49)	2.80±0.96	0.001
	Sometimes	84 (41.2)	2.40±0.89	
	Always	20 (9.8)	1.94±0.76	
Receive affective touch				
	Yes	102 (50)	2.98±0.88	0.001
	No	102 (50)	2.12±0.82	
IADLs				
	Dependent	142 (69.6)	2.58±0.92	0.343
	Independent	62 (30.4)	2.48±1.03	
Depressive symptoms				
	Normal	102 (50)	2.73±1.00	0.020
	Moderate	90 (44.1)	2.44±0.84	
	Severe	12 (5.9)	1.88±0.91	

Abbreviations: IADL, instrumental activities of daily living; SD, standard deviation.

In addition, the results showed that people who always lost words and thoughts when communicating with caregivers had a lower satisfaction score than people who never encountered this problem (1.94 *vs.* 2.80), with a significant difference between the two groups according to multiple pairwise comparisons using the Mann-Whitney U test. On the other hand, results showed that older adults who received affective touch from caregivers had higher satisfaction scores than those who did not receive this type of touch (2.98 *vs.* 2.12) (Table 3).

Regarding the psychological state, the analysis showed that people with severe depression have a lower average satisfaction score (1.88) compared to people without depression (2.73). The difference between the two groups was significant according to the paired comparisons made by the Mann-Whitney U test (p=0.020) (Table 3).

Table 4 presents the variables independently associated with the total satisfaction score of older adults with caregivers’ communication according to the multiple linear regression model. According to this table, the visual deficit is negatively associated with older adults’ satisfaction with caregivers’ communication (B=-0.276±0.12; p=0.029) and receiving affective touch from caregivers is positively associated with older adults’ satisfaction (B=0.745±0.12; p=0.001).

**Table 4:** Variables independently associated with total satisfaction score of older adults with caregivers’ communication according to the multiple linear regression model.

Variables	B±SE	β	p-value
Hearing impairment	0.120±0.140	0.061	0.392
Visual impairment	-0.276±0.125	-0.138	0.029
Lost words and thoughts	-0.192±0.104	-0.133	0.066
Receive affective touch	0.745±0.118	0.391	0.001
Depressive symptoms	0.141±0.103	0.090	0.171

Abbreviations: B, regression coefficient; SE, standard error; β, standardized coefficients. Note: NB: R2=0.333.

## DISCUSSION

The purpose of this study was to assess older adults’ satisfaction with caregivers’ communication skills and to identify influencing factors. Several authors have shown that communication skills include greeting, listening, respect, interest, using simple language, and answering questions^10,27^.

The results of this study showed that the dimensions of communication that had low satisfaction scores from older adults were those of answering questions, greeting, listening and interest. These results are consistent with a Japanese study that used the ABIM questionnaire and showed that patients give low scores to caregivers on answering questions and encouraging questions^28^ and also with an American study that showed that older patients are more likely to give low scores on different items of the ABIM questionnaire about caregivers’ communication skills^29^.

The low scores for greeting, answering questions, listening and interest explain the low total satisfaction score found in this study (2.55±0.95). This score is lower than the score of 3.64 found in a study conducted in Yemen^19^ and the score of 4.39 found in Kuwait^20^, while it is slightly higher than the score of 2.45 found in Saudi Arabia.^33^ This difference in scores could be explained by the different contexts of the studies and also by the age categories included in the survey. This study involved only adults aged 60 and over who have specific communication needs that differ from those of other age groups^14^, whereas studies in Yemen, Kuwait, and Saudi Arabia included other younger age groups.

In addition, patients’ satisfaction with caregivers’ communication could be influenced by several associated factors, namely patients’ sociodemographic and clinical characteristics. This study tried to determine the factors associated with older adults’ satisfaction with caregivers’ communication. The results showed that there was no association between the satisfaction of older adults and sociodemographic characteristics. These results are not consistent with a study conducted in primary health care centers, which showed that women show a lower satisfaction score than men and that this score is higher among people with a university education^20^. Similarly, these results do not agree with a study conducted in a hospital in Yemen and among patients of different age categories, which showed that patients’ satisfaction with caregivers’ communication is not associated with sex, while it is associated with age and education status^19^. This inconsistency in results could be explained by the context of the studies, whether it was a hospital or a primary health care center, and also by the age of the participants.

The results of this study, conducted in a hospital setting and among persons aged 60 years and older, showed that having a visual deficit and receiving affective touch from caregivers were the main factors associated with older adults’ satisfaction with caregivers’ communication.

Analysis of the results showed that older adults with visual impairment reported lower satisfaction than those without visual disorders. This finding is consistent with studies that have shown that people with visual impairment have difficulty interacting with caregivers, receive limited information, and receive minimal treatment from caregivers because caregivers are unaware of the specific needs of these individuals^21,34^. Visual impairment in older individuals can contribute to various morbidities, both physical, such as mobility limitations and dependence^35,36^, and mental, such as anxiety and depression^37–39^, as well as social ones, leading to feelings of loneliness and exclusion^40,41^. Caregivers must be aware of the impact of visual deficits on the various dimensions of the quality of life of older adults and must mobilize adequate and appropriate communication techniques to satisfy these people and provide them with quality care.

Results also showed that older adults who received affective touch from caregivers gave higher satisfaction scores to caregivers’ communication than those who did not receive such touch. This result corroborates several studies that have shown that affective touch facilitates communication and interaction between older adults and caregivers. These studies showed that the use of affective touch by caregivers during care calms older patients, encourages their participation, reduces the stress that accompanies caregiving, and thus promotes a good interpersonal relationship^42,43^. Furthermore, through touch, caregivers can demonstrate their interest and empathy towards elderly individuals, which can help build trust and facilitate the identification of their needs. Studies have shown that caregivers’ empathy enables them to accurately recognize expressions of sadness in older adults and can give them the ability to deduce potential care needs^44^.

Finally, this study has some limitations due to the fact that it was conducted in a hospital setting and could not be generalized. Further studies in primary healthcare centers would be very interesting.

In conclusion, older adults are not sufficiently satisfied with caregivers’ communication skills, particularly with regard to greeting, listening, interest and responding to questions. This satisfaction is influenced by several factors related to older adults, including the presence of visual impairment and the benefit of affective touch. People with visual impairments are more critical on the assessment, whereas those who have received affective touch from caregivers give higher scores to caregivers’ communication. Indeed, this study provides valuable insights into the importance of communication skills that healthcare professionals need to possess in order to offer quality care to the elderly. With the world’s population aging, it is essential that training centers for caregivers focus on specific techniques adapted to communicating with older adults. Caregivers need to be aware of the particular communication needs of the older people, especially those suffering from age-related health problems such as impaired vision. They must pay greater attention to greeting, listening to and questioning the older person, and emphasize the use of affective touch during care, to guarantee their satisfaction and provide them with quality care.
